# Coordination characterization of zinc metalloproteins

**DOI:** 10.1186/1471-2105-15-S10-P31

**Published:** 2014-09-29

**Authors:** Sen Yao, Robert M Flight, Hunter NB Moseley

**Affiliations:** 1 Department of Computer Engineering and Computer Science, University of Louisville, Louisville, KY 40292, USA; 2 Interdisciplinary Bioinformatics Program, University of Louisville, Louisville, KY 40292, USA; 3 Department of Molecular & Cell Biochemistry/ Markey Cancer Center/ Resource Center for Stable Isotope Resolved Metabolomics, University of Kentucky, Lexington, KY 40536, USA

## Background

Zinc metalloproteins play a key role in all three domains of life. Prior studies have explored the use of zinc coordination characteristics for the prediction of functions in metalloproteins. However, prediction methods have been hampered by the inaccuracy in characterizing the zinc coordination environment. In order to minimize the impact of this factor, we improved our method to better characterize zinc coordination.

## Materials and methods

We developed a systematic and fully automatic method to characterize the zinc coordination environment. Zinc metalloproteins were acquired from the worldwide Protein Data Bank (wwPDB). The overall zinc coordination environment was characterized in terms of coordination geometries, coordinating ligands, bidentation status, as well as side chain dihedral angles. In addition to the three major coordinations, tetrahedral, trigonal bipyramidal, and octahedral, we also examined four minor coordinations, trigonal planar, trigonal pyramidal, square planar, and square pyramidal. We calculated the number of binding ligands for each zinc site using criteria derived from an analysis of ligand-zinc bond lengths. Zinc sites with four, five, and six ligands were considered independently. K-means was used to differentiate the geometries based on angle statistics. Random forest was then applied to explore the interactions within different geometries.

## Results

We can automatically cluster the coordination of zinc metalloproteins into major and minor geometries. The consideration of four additional coordination geometries enables the detection of potential specific coordination errors like missing ligands within wwPDB entries. Bidentation usually introduces an abnormal small angle, which makes the coordination unsuitable for any of the theoretical geometries and thus should be examined separately. Random forest, which excels at integrating both categorical and non-categorical information, has revealed some interesting intra- and inter-cluster connections in our dataset. The better classification and characterization of zinc coordination should help us gain insight into specific functional tendencies of zinc metalloproteins.

